# First Report on the Ethnopharmacological Uses of Medicinal Plants by Monpa Tribe from the Zemithang Region of Arunachal Pradesh, Eastern Himalayas, India

**DOI:** 10.3390/plants6010013

**Published:** 2017-03-02

**Authors:** Tamalika Chakraborty, Somidh Saha, Narendra S. Bisht

**Affiliations:** 1 Institute of Ethnobiology, School of Studies in Botany, Jiwaji University, Gwalior 474011, India; tamalika.chakraborty@waldbau.uni-freiburg.de; 2 Chair of Site Classification and Vegetation Science, Institute of Forest Sciences, University of Freiburg, Tennenbacherstr. 4, D-79106 Freiburg, Germany; 3 Resource Survey and Management Division, Forest Research Institute, PO New Forest, Dehra Dun 248006, India; bishtnsifs@yahoo.com; 4 Chair of Silviculture, Institute of Forest Sciences, University of Freiburg, Tennenbacherstr. 4, D-79106 Freiburg im Breisgau, Germany; 5 Institute for Technology Assessment and Systems Analysis (ITAS), Karlsruhe Institute of Technology (KIT), Karlstr. 11, D-76133 Karlsruhe, Germany; 6 Directorate of Extension, Indian Council of Forestry Research and Education, PO New Forest, Dehra Dun 248006, India

**Keywords:** medicinal plants, traditional knowledge, Eastern Himalayas, mountain plants, ethnobotany, ethnopharmacology, bioprospecting

## Abstract

The Himalayas are well known for high diversity and ethnobotanical uses of the region’s medicinal plants. However, not all areas of the Himalayan regions are well studied. Studies on ethnobotanical uses of plants from the Eastern Himalayas are still lacking for many tribes. Past studies have primarily focused on listing plants’ vernacular names and their traditional medicinal uses. However, studies on traditional ethnopharmacological practices on medicine preparation by mixing multiple plant products of different species has not yet been reported in published literature from the state of Arunachal Pradesh, India, Eastern Himalayas. In this study, we are reporting for the first time the ethnopharmacological uses of 24 medicines and their procedures of preparation, as well as listing 53 plant species used for these medicines by the Monpa tribe. Such documentations are done first time in Arunachal Pradesh region of India as per our knowledge. Our research emphasizes the urgent need to document traditional medicine preparation procedures from local healers before traditional knowledge of tribal people living in remote locations are forgotten in a rapidly transforming country like India.

## 1. Introduction

The Himalayas are rich in diversity of medicinal plant species [1]. The culture of traditional healing of diseases using these plants is still prevalent among aboriginal mountain communities in the Himalayas. Arunachal Pradesh (approximately 84,000 km^2^ in size), a state belonging to the Republic of India, is situated in the Eastern Himalayas. The entire state is declared as a “biodiversity hotspot” with 5000 endemic flowering plant species as well as very high faunal diversity [1,2]. Also, this state is the home to 28 major tribes and 110 sub-tribes and is considered to be one of the most splendidly variegated and multilingual tribal areas of the world [3]. The traditional wisdom of healing among mountain tribal communities is orally transferred from one generation to the next generation through traditional healers, spiritual gurus, and elderly or sometimes ordinary people. This traditional wisdom, if not properly documented, can be lost by rapid modernization and religious reformation among mountain communities in Arunachal Pradesh where traditional customary practices are often regarded as a symbol of “*backwardness*” and “*unscientific*” by the educated and younger generations. Nevertheless, plant-based traditional wisdom inherited and carried forward to generation after generation in traditional communities has become a recognized tool in the search for new sources of drugs and pharmaceuticals in modern medicine [4]. Therefore, field based ethnobotanical and ethnopharmacological surveys to list medicinal plants and their uses are still relevant and worth the effort in order to bring out new clues for the development of drugs to treat human diseases [5]. 

Before coming to our research objectives, we would like to briefly mention the state of the art of ethnopharmacological research in the Himalayas. There are plenty of research works on the listing of the traditional uses of medicinal plants from the Himalayas. A search with the terms “medicinal plants * Himalayas” yielded 163 peer-reviewed articles listed in ISI Web of Knowledge on 20 February 2017. However, out of those 163 articles, 19 articles were found from the Eastern Himalayas and only two were on the Monpa tribe (please see Materials and Methods section for a detailed sociocultural description of the Monpa tribe). Haridasan et al., in the seminal works produced in 1998 and 1990, comprehensively listed medicinal and edible plants of the Monpa tribe and other tribes of Arunachal Pradesh [2]. Recently, Namsa et al. (2011) listed 50 plant species and recorded their ethnobotanical uses among people of the Monpa tribe at the southern range of their habitation (i.e., Kalaktang circle of West Kameng district of Arunachal Pradesh) [6]. These two publications provided general descriptions of the plants, traditional uses of the plants to cure certain diseases, and traditional ways of consumption of these plants or plant parts (e.g., pills, syrups, decoctions, etc.). Nevertheless, no ethnopharmacological studies have yet reported how, and in what proportion, multiple plant parts from different species can be used to prepare specific ethnomedicines for healing of diseases among the Monpa tribes or any other tribes of the Eastern Himalayas as per our literature research as of 20 February 2017. In addition, the traditional knowledges of the people of the Monpa tribe residing at their northern habitation range (i.e., Zemithang circle of Tawang district of Arunachal Pradesh) are still not adequately documented due to the remoteness of the location. 

Documentations of traditional ethnopharmacological know-hows are necessary for the preservation of traditional knowledges of Himalayan tribal communities. Such documentations could create interest among professional pharmacologists for the search of new medicines and motivate ethnologists to study high cultural diversity of the Eastern Himalayas of India. Those were the main motivations to carry out this research. This study aims to document traditional ethnopharmacological know-hows of medicinal drug making among Monpa people in the Zemithang region of the state of Arunachal Pradesh. 

## 2. Results

Our study was a notable departure from the previous studies from the area that mostly documented and described the use of plant parts in individual plant species. We documented and described 24 ethnomedicines prepared by traditional healers based on 53 species (Table 1). The medicines were comprised of 53 plant species of medicinal plants belonging to 21 families (Table 2). These traditional medicines were most commonly used to heal a wide range of diseases such as arthritis, rheumatic pain, malaria, cough and cold, dysentery, etc. In addition, we recorded descriptions of medicines for the treatment of diseases such as epilepsy (*Pambrey*), herpes (*Bukbukpa-khaksa-chandongbra*), and oedema (*Darshek sheng nye putpoo*) that have rarely been reported in past studies. Our main result is presented in Table 1 which provides a list of ethnomedicines and their preparations by traditional ethnopharmacological techniques. 

## 3. Materials and Methods 

### 3.1. Sociocultural Description of the People from the Monpa Tribe

The Monpa people are a Buddhist tribe belonging to the Mahayana (Tibetan–Lamaist) *Gelukpa* and *Nyngmapa* sect. The Monpa people are inhabitants of the western most districts of the Tawang and West Kameng regions of Arunachal Pradesh, India. Their main centers of habitation are in and around the administrative headquarters of Zemithang, Tawang, Dirang, and Kalaktang. Depending on the place of living and the geographical location of these centers, they are often called as Zemithang-Tawang or “Northern Monpas”, Dirang or “Central Monpas”, and Kalaktang or “Southern Monpas”. The language used by Dirang and Kalaktang Monpa are different from that of Tawang Monpas. Dirang and Kalaktang Monpas use a dialect of Bhutanese Brokpa language, whereas Zemithang-Tawang Monpas use a dialect of Tibetan-Bhutanese Dakpa language. However, many other aspects of their life are quite similar. In Dakpa language, the name “Mon” and “Pa” signify the “Men of the Lower Country” or the inhabitants of southern regions to Tibet. 

The Monpa villages are often situated on the slopes of the hills or in the valleys. A striking characteristic of the Monpa villages is the presence of a “*Gompa*” (Buddhist village monastery), often situated on the top of the hill and surrounded by prayer flags (“*phan*”), stone shrines (“*mane*”), and small chapels called “*chorten*” which are often found alongside the roads and foot-lanes. The houses are usually double or triple storeyed, and made mainly of locally sourced stone. Each house has a family chapel with a wooden, stone, or brass statue of the *Lord Buddha*.

The adornments and clothing are diverse and colourful. People cover their whole bodies with a variety of well-designed woolen garments. The women do the traditional spinning and weaving of the garments, as well as carpet making. The Monpa people can be recognized from a long distance owing to the attractive color of their clothing, which is a mellow strawberry red. The Monpa people love this color and dye their clothes themselves using the locally available natural dyes from diverse species of Rhododendrons and other plants. They love music and dance. Their musk-dances are very famous and attract a large number of tourists. The “Losar” or the Buddhist New Year is the most important festival celebrated among them, which is organized in February. Monpa villages could be located at a great distance by their high fluttering Buddhist prayer flags on which is printed in Tibetan script “*Om Mani Pame Hung*” which means “*Hail to him who is born as a Jewel in a Lotus*”. 

The Monpa people typically eat various types of locally grown vegetables, which are often cultivated by using tradtional methods [7]. Drinking yak milk, making homemade butter and dry cheese from yak milk (e.g., the famous “*churpi*” dry cheese), eating yak meat, pork, chicken, mutton, cultivation of multiple species of cereal and pulse through sustainable mountain agriculture based on tradtional ecological knowledges without any use of pesticides, herbicides, and chemical fertilizers are common practice [7]. Monogamy appears to be the form of marriage followed by the Tibetan Buddhist traditions. Tattooing is not typically observed among Monpa people, which is a stark contrast to the people from other tribes such as the *Nishi* and *Adi* in nearby districts. Information relating to the origin and migration of the Monpa people to their present habitat in Arunachal Pradesh is largely obscure. This is because written records on the history of Monpa people from the middle ages or beyond are very rare. Thus, it remains a matter of further anthropological and archeological research to find out the route and approximate time of their relationship with either the Tibetans or Bhutanese, or even with the people of Pan-Indian origin. When we visited Namshu village in Dirang region, the “*Gaobura*” or the village headman told us a folklore story about a marriage between a prince from Bhutan and a local Monpa girl from that village. The story indicates the Bhutanese influence among Dirang Monpa. The language of the Eastern Bhutan and Dirang areas are similar. Here we can quote from von Fürer-Haimendorf of Austria who was the most prominent anthropologist that ever worked with the tribes of this region [8]: “*THE REGION, WHICH ADJOINS TO THE WEST OF THE MOUNTAIN KINGDOM OF BHUTAN, DIFFERS FROM THE REST OF ARUNACHAL PRADESH BOTH TOPOGRAPHICALLY AND CULTURALLY. WHEREAS, ELSEWHERE NATURE AND THE TERRAIN HAD PREVENTED THE DEVELOPMENT OF CARAVAN ROUTES SUITABLE FOR PACK ANIMALS IN THE WESTERNMOST PART OF ARUNACHAL PRADESH. HOWEVER, WHERE THE CLIMATE AND GEOGRAPHICAL CONDITION WERE FAVORABLE, THE TRADE ROUTES WERE OPENED LINKING THE TERRITORY BOTH WITH TIBET AND THE PLAINS OF ASSAM OF INDIA. HENCE CONDITIONS ARE SIMILAR TO THOSE PREVAILING IN BHUTAN AND FURTHER WEST TO SIKKIM AND NEPAL. ALONG WITH THESE TRANS-HIMALAYAN TRADE ROUTES, TIBETAN CULTURAL ELEMENTS AND ULTIMATELY BUDDHIST MONKS AND NUNS INFILTRATED INTO THE MOUNTAIN REGION LYING BETWEEN THE EASTERNMOST OF BHUTAN AND THE SOUTHERN BORDER OF TIBET.*” 

### 3.2. Study Area

**Figure 1:**
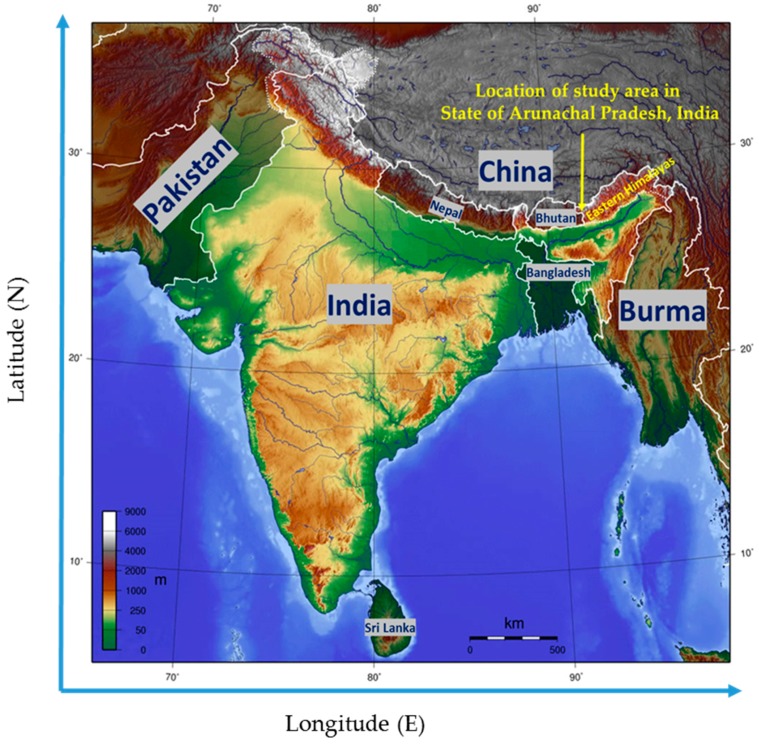
Location of the study area at the Zemithang region in the state of Arunachal Pradesh, India pointed by the yellow arrow (map not to scale).

The study area is located in the extreme north of the north-western Arunachal Pradesh. The areas of investigations are situated at the Lumla–Zemithang administrative circle of the Tawang district of Arunachal Pradesh (Figure 1). This region is situated along the bank of the river Namshyang Chu that flows through the area. The name, exact locations, and altitude of the three villages where the study took place are as follows: (1) village Kublaitang (27°37′070″ N, 91°41′618″ E, elevation 2224 m); (2) village Shakti (27°36′736″ N, 91°42′970″ E, elevation 2020 m); and (3) village Lumpho (27°43′140″ N, 091°43′069″ E, elevation 2550 m). The research areas fall under the middle Himalayan range of the Eastern Himalayas. The soil on the hills is moderately deep and moist, fertile loamy layer stained with humus. At places, shallow soils are not uncommon with underlying boulders and rocks. The subsoil at lower elevations consists of mostly boulders and pebbles superimposed by a layer of a sandy loam of various depths with layers of humus overtop. The relative humidity of this area varies from 30% to 80%. Southern aspects at low altitude areas are more humid than any other places in the region. The annual temperature in this area varies from −10 degrees Celsius to +15 degrees Celsius. The area typically receives 1500–1800 mm rainfall every year. The dry months are December, January, and February. The pre-monsoon rainfall starts from the end of the March. Highest rainfall is observed in June, July, and August [9]. The forest type of the research area is the Northeastern Himalayan subalpine mixed conifer forests. The top canopy of the forest consists of *Abies densa*, *Juniperus wallichiana*, *Illicium griffithi*, *Pinus wallichiana*, *Quercus* spp., and *Cupressus torulosa*. The secondary canopy layer mainly consists of *Rhododendron* spp., *Betula utilis*, *Pyrus aucuparia*, and *Salix wallichiana*. The trees of the forest ground storey are dominated by *Juniperus recurva*, *Cassiope fastigiata*, and *Rhododendron* spp. [10].

### 3.3. Field Surveys

Field surveys were carried at the three sample villages of the Zemithang region of Tawang district. The research was carried out in three stages. In the first stage, ethnobotanical data were collected from the research area. At the second stage, ethnopharmacological information was collected from the same research area. The herbariums of the collected plant specimens were prepared and verified at the third stage of the research at the Forest Research Institute of Dehradun, India. The field identifications of the plants were mostly done by using field guide with colored photographs of the plants by Polunin and Stainton [11]. Some unidentified and partially identified plants from the field were brought to the specialists at the Forest Research Institute of Dehradun, India for full identification. The participatory transect walk, interview, and discussions with traditional healers were used for ethnobotanical data collection. The total number of participatory transects established were three for every village, resulting in a total of nine across all three villages. The length of each transect was 2 km from the center of the village to three different outward directions, depending on aspects of the village. We used three different groups for the transect walks. These groups were common village people including men and women, hunters, and traditional healers. Two walks with every group with different people were conducted. As such, the total number of transect walks per village was six, thereby totaling 18 transect walks across all three villages. This type of data collection design was followed for the robustness of ethnobotanical information. Apart from this technique for collecting plant specimens with ethnobotanical values, we used a structured questionnaire for interviews and group discussions regarding the ethnopharmacological techniques of medicine preparation for the collected plants. The people who participated in transect walks were not selected for questionnaire surveys in order to avoid repetition and establish a more general idea among larger population groups. The participatory transect walks were mostly carried out in spring and summer when a large flush of herbaceous plants grow in the forest, pasturelands, and meadows after the melting of winter snow. At the second stage of the research, ethnopharmacological information was collected from the high ranked monks and traditional healers who prepare medicine from plants for the healing of the tribal people. In each village, we selected at least three independent healers or monks for this purpose. After gathering the information, we performed a qualitative assessment for reaching a consensus among the respondents and rejected the conflicting responses. The basic information that was collected from these monks and traditional healers were regarding (1) the plants needed to make medicine; (2) the use of plant parts; (3) the different ratios of plant use; (4) the techniques of preparation; (5) the doses and prescription to the patients; and (6) the medicinal uses. The third stage of the research was carried out at the Resource Survey and Management Division of the Forest Research Institute, Dehradun, India. Taxonomical classification was performed with the help of the Botany Division of the Forest Research Institute, and identified plant specimens were confirmed by using the herbaria of the same division for comparison purposes. The specimens with detailed taxonomic information, name of collectors, and place and date of collections were finally deposited to The Course Coordinator of Postgraduate Programs, Forest Research Institute University (Dehra Dun, India) for future references. We had received permission from the local forest authorities in addition to having obtained consents from the traditional healers before doing this survey. 

## 4. Discussion

The list of plant species and utilization of plant parts for different diseases documented in this study support a recent study carried out by Namsa et al. (2011) on the southern or Kalaktang Monpas [6]. The list we provided for the medicinal plants is not completely new to ethnobotanists, as it was already listed in old research works on medicinal plants of the Himalayas (see [12,13,14]). This proves that the plants we listed are already confirmed as “medicinal plants” by past researchers from the other parts of the Himalayas. However, the detailed ethnopharmacological descriptions or traditional ways of preparation for the herbal drugs and medicines were rarely documented. Due to this reason, a search with the terms “ethnopharmacology * Himalayas” yielded only three articles on 20 February 2017 in the ISI Web of Knowledge. For example, Gangwar et al. [15] worked on ethnopharmacological uses of *Mallotus philippinensis* Muell. Arg, and Stobdan et al. [16] did a similar work on *Hippophae rhamnoides* L. We found only one article, a study by Abbasi et al. [17], that was similar to our study and described ethnopharmacological knowledges of medicine preparation from a Himalayan region of the Pakistan Himalayas. Therefore, we emphasize that this is the first documented study on ethnopharmacology of a tribe from Arunachal Pradesh. We assume that in most cases modern pharmacologists and researchers start chemical assessments of the medicinal plants without giving much attention to the traditional ways of drug preparation by the tribal communities. This could be the reason behind the high number of studies on ethnobotany of medicinal plants from the Himalayas, but the comparatively minimal number of studies on ethnopharmacology. In South India, the Kani tribe uses similar approach for traditional medicine making [5], which supports the notion that tribal healers do use certain systematic techniques for drug preparation. The ethnopharmacological knowledge of traditional healers are generally transferred orally to the next generation, thus, making the knowledge vulnerable to being forgotten or lost.

In this context, we would like to provide a few examples of past pharmacological studies that had reported similar utilization of some medicinal plants listed in this study. Ghildiyal et al. (2012) showed that ethanolic extracts from *Hedychium spicatum* can inhibit respiratory as well as gastrointestinal disorders in rats and guinea pigs [18]. We showed in this study that the ethnomedicine *Blenga* prepared from the same plant was used for the treatments of dysentery and chest pain. In 2007, Nazir et al. extracted a drug called “Bergenin” from the species *Bergenia stracheyi* and proved that this drug can be used to treat arthritis in mice [19]. Interestingly, we found that an ethnomedicine named as *Bragen* (prepared from *Bergenia stracheyi* as well) was also used for the treatment of arthritis. Recently in 2014, Kumar et al. reported that the extracts of *Houttuynia cordata* can be used for the healing of hemorrhoids, and this species is frequently used in tradtional Tibetan and Chinese medicines [20]. We found that the ethnomedicine *Maraptang* prepared from *Houttuynia cordata* were also used by the tradtional Monpa healers for the treatment of piles which is a type of hemorrhoid. These examples mentioned above showed that the tradtional ethnomedicines used by the healers of Zemithang Monpa may have some potential to cure or manage some diseases. However, detailed pharmacological studies are needed to evaluate the potential of these medicines. A study by Witt et al. (2009) in Sikkim and Eastern Nepal (also part of the Eastern Himalayas) comprehensively listed 138 species of plants from tropical to alpine regions of the Himalayas used specifically in Tibetan medicine [21]. The majority of the species listed in our study were also reported by Witt et al., but detailed descriptions of the preparations for the ethnomedicines were not provided. 

The results of this study should be interpreted very cautiously. The traditional ethnopharmacological knowledge of the Zemithang-Monpa tribe presented here for some diseases must not be treated as a general prescription under any circumstances, as scientific trials have not been undertaken nor the “traditional ethnomedicines” have ever been certified by any governmental authority such as the Central Drugs Standard Control Organization of India. There is also a high probability that the descriptions presented here may not be the same throughout the study region. Nevertheless, our main goal was not to certify or validate traditional medicines, but rather to document the uses and preparation of traditional medicines used by tribal people. The field method applied for data collection (i.e., participatory transect walk) also had some limitations. This method was helpful in remote regions where time and logistics are always a constraint of field work. Nevertheless, future research should establish more sample plots and cover larger regions in order to list more medicinal plants. 

## 5. Conclusions 

We have documented for the first time the vernacular names combined with ethnopharmacological preparations of ethnomedicines among Monpa tribes from the Zemithang region of Arunachal Pradesh, India. Past studies on ethnobotany in the Arunachal Pradesh, Eastern Himalayas, had listed uses of medicinal plants, however, we found that traditional healers use diverse species and plant parts in specific proportions for drug preparations. Our study illustrates the diversity of medicinal drug preparations and traditional knowledge that has passed through generation after generation of Monpa people. The ethnopharmacological documentation presented in this study should motivate researchers to carry out further scientific work on pharmacology, bioprospecting, and the cultivation of medicinal plants for the socioeconomic development in the region. Under ongoing warming of the Himalayas and mass migration of people from the mountain areas to cities, our study also highlights the need to document the traditional knowledge regarding the use of local flora and to develop strategies to conserve them before the traditional knowledges are lost or forgotten.

## Figures and Tables

**Table 1:** List of 24 ethnomedicines used by the Zemithang Monpa people and the associated medicinal plants documented in this study.

Number	Name of the Ethnomedicines (in Monpa Tribal Language of Zemithang Dialect)	Type of Medicines	Name of Medicinal Plants Used for Ethnomedicines	Proportion of Used Plant Parts (*Bray* in local language is a Buddhist prayer bowl. It could be made of gold, silver, brass, copper, stone, or wood and is often used for religious offerings. 1 *bray* can contain approximately 900 g of grain).	Mode of Preparations	Medicinal Uses
1	Arkadamasisi	paste	*Crawfurdia speciosa* Wall.	1/4 bray of dried root + 1/8 bray of dried flower	dried roots and flower crushed together to prepare powder and then mixed with water to prepare paste	paste is applied externally for healing wounds
2	Baribama	decoction	*Aristolochia griffithii* Hook.f.	1/8 bray of raw washed roots	roots are boiled with water to prepare a decoction	decoction is taken as blood purifier and purgative
3	Blenga	pills	*Hedychium spicatum* Buch.-Ham.	1 bray of washed raw roots	raw roots are crushed and small round pills are prepared and sun dried	pills are taken orally for treatment of dysentery, chest pain, cough and cold
4	Bomdeng	paste	*Cirsium falconeri* Hook.f., *Cirsium verutum* D. Don and *Onopordum acanthium* L.	1/2 bray of washed raw root of *C. falconeri* + 1/2 bray of washed raw root of *C. verutum* + 3/4 bray of washed raw root of *O. acanthium*	raw roots are mixed together and crushed to prepare paste	paste is applied externally to treat arthritis
5	Bragen	syrup	*Bergenia stracheyi* Hook.f. & Thorns.	1 bray of washed fresh leaves	clear fresh leaves are crushed to prepare paste and mixed with 1/4 bray local millets wine to prepare syrup	syrup is taken for treating rheumatic pains
6	Bukbukpa-khaksa-chandongbra	paste	*Campanula latifolia* Linn., *Codonopsis* *clematidea* Schrenk. and *Codonopsis viridis* Wall.	1/2 bray of washed fresh leaves and 1/4 bray of fresh flowers of each species + 1/4 bray of conch powder + 1/4 bray of water	leaves and flowers are crushed together and mixed with conch powder and water to prepare paste	paste is applied externally to treat herpes
7	Chandoo-konghlin-bhor	powder	*Aconitum ferox* Wall. ex Ser., *Aconitum heterophyllum* Wall. ex Royle, *Aconitum hookeri* Stapf., *Geranium polyanthes* Edgeworth & J. D. Hooker, *Geranium wallichianum* D. Don and *Picrorhiza kurrooa* Royle ex Benth.	1 small dried root from each plants of *A. ferox*, *A. heterophyllum* and *A. hookeri* (total 5 g mixture of three plants) + 3 bray of dried root of *G. polyanthes* and *G. wallichianum* + 1 bray of dried root *P. kurrooa*	all ingredients are mixed together and crushed to prepare a powder	powder is taken orally to overcome poisoning effects
8	Chhalachhusar	syrup	*Meconopsis grandis* Prain and *Meconopsis paniculata* D. Don	1/2 bray of dry leaves from each plant + 1/2 bray of dy flowers from each plant	dried leaves and flowers are mixed together and crushed to prepare powder, and a small amount of powder (5 g) is mixed with 1 bray of water to prepare a syrup	syrup is taken to treat sexually transmitted diseases
9	Chhurchu doho keusheng	pills	*Rheum australe* D. Don, *Rheum nobile* Hook.f. & Thoms. and *Bistorta affinis* D. Don	1/2 bray of fresh roots from each species + 1/4 bray of dried flowers from each species	fresh roots and dried flowers are crushed together to make a paste, then small round pills are prepared and sun dried	pills are taken orally to overcome poisoning effects
10	Comrep	syrup	*Rubus ellipticus* Smith and *Rubus paniculatus* Smith	1/2 bray of fresh ripe fruits from each plant	roots are mixed together and crushed to prepare a thick syrup	syrup is used for treatment of cold and cough
11	Darshek sheng nye putpoo	decoction	*Pieris formosa* (Wallich) D. Don; *Vaccinium nummularia* Hook.f. & Thoms.	1/4 bray of fruits of *P. formosa* + 1/4 bray of fruits of *V. numularia* + 1/2 bray of fresh roots of *P. formosa* + 1/2 bray of fresh roots of *V. numularia*	mixture of all fresh fruits and roots along with water is boiled to prepare a decoction	decoction is taken to cure oedema
12	Dhamrep	paste	*Fragaria nubicola* Lindl., *Geum elatum* Wall. and *Potentilla peduncularis* D. Don.	1/2 bray of *F. nubicola* fresh fruits + 1/8 bray of dried roots of *G. elatum* + 1/4 bray of leaves of *P. peduncularis*	fresh fruits, leaves, and dried roots are crushed together to prepare a paste	paste is taken orally to treat cold, cough, and fever
13	Gin sheng	powder	*Panax pseudoginseng* Wall.	1/4 bray of dried rhizomes	dried rhizomes are crushed to prepare powder, which is taken with water	used for treating depression and fatigue
14	Karpo Chiito	paste	*Iris clarkei* Baker	1/4 bray of dried flower, leaves, stem, and root	dried flowers, leaves, stem parts, and roots are crushed together to prepare powder and mixed with local millets wine to prepare paste	paste is used externally to treat muscle pain
15	Lowa bur	pills	*Lomatogonium carinthiacum* (Wulfen) Rchb.	1/4 bray of dried roots	dried roots are crushed and small round pills are prepared and sun dried	pills are taken orally to treat cold, cough, and fever
16	Maraptang	pills	*Houttuynia cordata* Thunb.	1/4 bray of dried roots	dried roots are crushed and small round pills are prepared and sun dried	pills are taken orally for treatment of piles
17	Nyasheng jormu	paste and pills	*Viscum articulatum* Burm. f.	1/4 bray of fresh roots + 1/4 bray of fresh leaves + 1/4 bray of fresh stems	fresh roots, leaves, and parts of stem are crushed together to prepare paste; sometimes paste is used to prepare small round pills and sun dried	paste is used to join broken bones, treating pain from swelling of nerves and healing wounds; pills are used for treatment of infertility among women
18	Pambrey	mixture	*Anaphalis monocephala* DC., *Anaphalis triplinervis* Sims., *Gnaphalium hypoleucum* DC., *Leontopodium himalayanam* DC., *Leontopodium jacotianum* Beauv., *Tanacetum tibeticum* Hook.f. and *Tanacetum gracile* Hook.f. & Thoms.	1/2 bray of flowers from each of the plants	flowers are kept in a dark place for two days after plucking and then mixed together	used to treat epilepsy, mildly warm mixtures are applied on the bare head of the patient (two times a day) consecutively for 15 to 20 days
19	Pangen	pills	*Gentiana depressa* D. Don, *Gentiana ornata* Wallich ex G. Don, *Gentiana phyllocalyx* C. B. Clarke and *Gentiana tubiflora* Wallich ex G. Don.	1/4 bray of dried roots from each of the plants	dried roots are crushed and then mixed with 1/4 bray of local millet wine and 1/2 bray of water and small round pills are prepared and sun dried	pills are used to treat cough, cold, and headache
20	Rah-nya	decoction	*Smilacina purpurea S. oleracea* and *Polygonatum multiflorum* Allem.	1/4 bray of fresh roots from each of the plants	roots are boiled with water to prepare a decoction	is used for the treatment of malaria
21	Rambhoo tsarphakur	paste	*Morina longifolia* Wall., *Pterocephalus hookeri* (C. B. Clarke) Hock.	1/4 bray of dried flowers, 1/2 bray of fresh roots, 1/4 bray of fresh fruits of *M. longifolia* + 1/8 bray of dried flower, 1/2 bray of fresh roots, 1/2 bray of fresh fruits of *P. hookeri*	flowers, roots, and fruits of both plants are mixed together and crushed to prepare paste	paste is applied for healing chest pain
22	Trahm-Sheng	paste	*Corydalis cashmeriana* Royle.	1/4 bray of fresh leaves + 1/4 bray of fresh flower	fresh leaves and flowers are crushed to prepare paste	paste is applied for healing wounds
23	Wang La	powder	*Swertia chirayita* (Roxb. ex Flem.) Karst. and *Swertia hookeri* C. B. Clarke	1/2 bray of dried whole plants	dried whole plants are crushed to prepare powder, which is taken with water	powder is used to treat malaria, and is also used as a purgative and laxative
24	Whan	pills	*Lilium nepalense* D. Don	1/2 bray of dried roots	dried roots are crushed and mixed with water to prepare small round pills which are then sun dried	pills are used for treating gastritis and stomachic

**Table 2:** List of recorded plants used in Ethnomedicine.

Serial Number	Botanical Name	Family	Type
1	*Aconitum ferox* Wall. ex Ser.	Ranunculaceae	herb
2	*Aconitum heterophyllum* Wall. ex Royle	Ranunculaceae	herb
3	*Aconitum hookeri* Stapf.	Ranunculaceae	herb
4	*Anaphalis monocephala* DC.	Compositae	herb
5	*Anaphalis triplinervis* Sims.	Compositae	herb
6	*Aristolochia griffithii* Hook.f.	Aristolochiaceae	vine
7	*Bergenia stracheyi* Hook.f. & Thorns.	Saxifragaceae	herb
8	*Bistorta affinis* D. Don	Polygonaceae	herb
9	*Campanula latifolia* Linn.	Campanulaceae	herb
10	*Cirsium falconeri* Hook. f.	Asteraceae	herb
11	*Cirsium verutum* D. Don	Asteraceae	herb
12	*Codonopsis clematidea* Schrenk.	Campanulaceae	herb
13	*Codonopsis viridis* Wall.	Campanulaceae	herb
14	*Corydalis cashmeriana* Royle.	Papaveraceae	herb
15	*Crawfurdia speciosa* Wall.	Gentianaceae	herb
16	*Fragaria nubicola* Lindl.	Rosaceae	herb
17	*Gentiana depressa* D. Don	Gentianaceae	herb
18	*Gentiana ornata* Wallich ex G. Don	Gentianaceae	herb
19	*Gentiana phyllocalyx* C. B. Clarke	Gentianaceae	herb
20	*Gentiana tubiflora* Wallich ex G. Don.	Gentianaceae	herb
21	*Geranium polyanthes* Edgeworth & J. D. Hooker	Geraniaceae	herb
22	*Geranium wallichianum* D. Don	Geraniaceae	herb
23	*Geum elatum* Wall.	Rosaceae	herb
24	*Gnaphalium hypoleucum* DC.	Asteraceae	herb
25	*Hedychium spicatum* Buch.-Ham.	Zingiberaceae	herb
26	*Houttuynia cordata* Thunb.	Saururaceae	herb
27	*Iris clarkei* Baker	Iridaceae	herb
28	*Leontopodium himalayanam* DC.	Asteraceae	herb
29	*Leontopodium jacotianum* Beauv.	Asteraceae	herb
30	*Lilium nepalense* D. Don	Liliaceae	herb
31	*Lomatogonium carithiacum* (Wulfen) Rchb.	Gentianaceae	herb
32	*Meconopsis grandis* Prain	Papaveraceae	herb
33	*Meconopsis paniculata* D. Don	Papaveraceae	herb
34	*Morina longifolia* Wall.	Dipsacaceae	herb
35	*Onopordum acanthium* L.	Asteraceae	herb
36	*Panax pseudoginseng* Wall.	Araliaceae	herb
37	*Picrorhiza kurrooa* Royle ex Benth.	Scrophulariaceae	herb
38	*Pieris formosa* (Wallich) D. Don	Ericaceae	shrub
39	*Polygonatum multiflorum* Allem.	Convallariaceae	herb
40	*Potentilla peduncularis* D. Don.	Rosaceae	herb
41	*Pterocephalus hookeri* (C. B. Clarke) Hock.	Dipsacaceae	herb
42	*Rheum australe* D. Don	Polygonaceae	herb
43	*Rheum nobile* Hook.f. & Thoms.	Polygonaceae	herb
44	*Rubus ellipticus* Smith	Rosaceae	shrub
45	*Rubus paniculatus* Smith	Rosaceae	shrub
46	*Swertia chirayita* (Roxb. ex Flem.) Karst.	Gentianaceae	herb
47	*Smilacina oleracea* (Baker) Hook.f.	Liliaceae	herb
48	*Smilacina purpurea* (Wall.) H.Hara	Liliaceae	herb
49	*Swertia hookeri* C. B. Clarke	Gentianaceae	herb
50	*Tanacetum gracile* Hook.f. & Thoms.	Asteraceae	herb
51	*Tanacetum tibeticum* Hook.f.	Asteraceae	herb
52	*Vaccinium nummularia* Hook.f. & Thoms.	Ericaceae	shrub
53	*Viscum articulatum* Burm. f.	Viscaceae	shrub
